# In silico analysis of intestinal microbial instability and symptomatic markers in mice during the acute phase of severe burns

**DOI:** 10.1186/s12866-024-03266-9

**Published:** 2024-04-15

**Authors:** Bochen Hou, Honglan Zhang, Lina Zhou, Biao Hu, Wenyi Tang, Bo Ye, Cui Wang, Yongmei Xu, Lingyun Zou, Jun Hu

**Affiliations:** 1 Department of Neurology, Southwest Hospital, Third Military Medical University (Army Medical University), Chongqing, 400038 China; 2 Department of Clinical Data Research, Chongqing Emergency Medical Center, Chongqing Key Laboratory of Emergency Medicine, Chongqing University Central Hospital, School of Medicine, Chongqing University, Chongqing, 400014 China; 3 School of Computer Science, Chongqing University, Chongqing, 400030 China; 4 Chongqing University of Technology, Chongqing, 400054 China

**Keywords:** Severe burn, Acute phase, Gut microbiome, Machine learning, Inflammatory

## Abstract

**Background:**

Severe burns may alter the stability of the intestinal flora and affect the patient’s recovery process. Understanding the characteristics of the gut microbiota in the acute phase of burns and their association with phenotype can help to accurately assess the progression of the disease and identify potential microbiota markers.

**Methods:**

We established mouse models of partial thickness deep III degree burns and collected faecal samples for 16 S rRNA amplification and high throughput sequencing at two time points in the acute phase for independent bioinformatic analysis.

**Results:**

We analysed the sequencing results using alpha diversity, beta diversity and machine learning methods. At both time points, 4 and 6 h after burning, the Firmicutes phylum content decreased and the content of the Bacteroidetes phylum content increased, showing a significant decrease in the Firmicutes/Bacteroidetes ratio compared to the control group. Nine bacterial genera changed significantly during the acute phase and occupied the top six positions in the Random Forest significance ranking. Clustering results also clearly showed that there was a clear boundary between the communities of burned and control mice. Functional analyses showed that during the acute phase of burn, gut bacteria increased lipoic acid metabolism, seleno-compound metabolism, TCA cycling, and carbon fixation, while decreasing galactose metabolism and triglyceride metabolism. Based on the abundance characteristics of the six significantly different bacterial genera, both the XGboost and Random Forest models were able to discriminate between the burn and control groups with 100% accuracy, while both the Random Forest and Support Vector Machine models were able to classify samples from the 4-hour and 6-hour burn groups with 86.7% accuracy.

**Conclusions:**

Our study shows an increase in gut microbiota diversity in the acute phase of deep burn injury, rather than a decrease as is commonly believed. Severe burns result in a severe imbalance of the gut flora, with a decrease in probiotics and an increase in microorganisms that trigger inflammation and cognitive deficits, and multiple pathways of metabolism and substance synthesis are affected. Simple machine learning model testing suggests several bacterial genera as potential biomarkers of severe burn phenotypes.

**Supplementary Information:**

The online version contains supplementary material available at 10.1186/s12866-024-03266-9.

## Introduction

A severe burn is a serious trauma that can pose a serious risk to personal safety. Scalding can lead to the destruction of skin tissue, which can cause infection, blood loss, shock and other complications. In addition, burns can cause scarring, deformity, and dysfunction, all of which may have long-term effects on the patient’s physical and mental health [1]. Burns also cause neurological cognitive dysfunction, including memory deficits, amnesia, dementia, depression, anxiety, post-traumatic stress disorder (PTSD), hallucinations and delirium [2].

The human gut microbiome is a complex community system of over 100 trillion bacteria that defends against pathogenic microorganisms and helps to maintain the intestinal epithelial barrier [3]. Metabolites of the intestinal microbiota, including some components from bacterial cells, are mainly produced in the vicinity of intestinal epithelial cells. They are deeply involved in a small group of shared pathways that control intestinal barrier function, including maintenance of energy homeostasis, regulation of osmotic homeostasis, regulation of biofilm formation, and others. Dysbiosis affects the homeostasis of metabolites, which impairs the function of the intestinal barrier [4, 5]. Recently, the gastrointestinal bacterial microbiome has been shown to be a key component in regulating the immune response and recovery from burn injury, and may also contribute to significant adverse sequelae after injury, such as sepsis and multiple organ failure [6]. There is clear evidence that microbial communities are significantly altered after severe burn injury, but these changes are difficult to observe directly from the body or to correlate with changes in disease state. Therefore, mice have been used to model severe burn injury to study the changing characteristics of the gut flora and their potential effects. Studies in the CF-1 mouse model of full-thickness burn confirmed differences in gut microbiology after burn injury and identified 38 pathways that were differentially expressed between sham-burned and burned mice, including bacterial invasion of epithelial cells and gap and adherens junction pathways [7]. Another study using the C57BL/6 mouse model found an increase in intestinal permeability after severe burns, which peaked at 6 h post-burn and was approximately 20-fold higher than that of controls, in addition to a significant alteration in the expression of tight junction proteins, a significant decrease in the content of short-chain fatty acids, and the presence of up-regulation of inflammatory factors, suggesting that the disruption of the intestinal barrier is associated with an alteration in the intestinal microbial community [8]. Notably, analysis of sequencing data from gut microbiota samples at different time points after burn injury revealed that the abundance of the gut microbiota began to decrease from day 7 after burn injury, and its major components and microbial community structure also changed over time. A study from mice showed that by the day 28 after burn injury, the composition of the microbiome had essentially returned to the pre-burn levels [9]. Similar bacterial diversity was also found between the pre- and post-burn groups in the rat model, but the composition of the gastrointestinal microbiota changed after burns [10].

The current situation is that less progress has been made in studying the relationship between severe burns and gut microbes in the mouse model, particularly in the acute phase within 1 day of the burn. Not only do we know little about the changing characteristics of gut microbes during the acute phase, but there is an even greater lack of in-depth exploration of which are the key microbes associated with acute phase symptoms and what role they may play in disease progression. Accurate answers to these questions, which can be corroborated by studies in humans [11], may help to explain the origin and subsequent development of infections, as well as to infer the prognosis of burn patients [12, 13]. Once the differences between the different samples are known, it means that their characteristics can be extracted. However, the mechanisms of association between traits and phenotypes or outcomes are often unexplained and involve a great deal of experimental evidence. Machine learning models are independent of this evidence. They can be used well to distinguish phenotypes or predict outcomes by simply calculate features from training data to train the model, and a variety of algorithms are included that can be adapted to datasets of varying sizes. As a result, machine learning models are widely used in predictive tasks related to the gut microbiota [14].

In this study, we established a mouse model of deep III degree burns, collected intestinal contents for microbiome sequencing, and analysed the characteristics of microbial community changes in the absence of burns and at 4 and 6 h after burns using in silico methods to search for key microbial species that can indicate the acute phase of burns. The potential value of microbial applications to diagnose the extent of burn injury was explored by building machine learning models.

## Materials and methods

### Animals

Twenty-four healthy adult female C57BL/6 mice were purchased and randomly divided into a burn group (16 mice) and a control group (8 mice), and experiments were performed after 1 week of acclimation in a room with constant temperature (25 ± 2 °C) and 12-hour light/dark cycles. One day before the establishment of the burn model, the mice were anaesthetised by intraperitoneal injection of 1% sodium pentobarbital (7–8 ul/g), and the hairs on the back were plucked and washed off after uniform application of depilatory cream, and then housed in separate cages. On the following day, mice were anaesthetised and then burned on the exposed skin (95 °C, 9 S) using a heat-stable scalding apparatus to produce a full-thickness burn with 30–50% total body surface area. Burn-wounded mice were randomly divided into two groups, supplemented with 0.9% normal saline and placed on a thermostatic pad to keep warm, and then the intestinal contents were collected at 4 and 6 h for 16s rRNA sequencing, respectively.

This animal study was approved by the Animal Protection and Use Committee of the Third Military Medical University (Army Medical University), and all protocols were approved by the Medical and Ethics Committee of the Southwest Hospital of the Third Military Medical University (Army Medical University), Chongqing, China.

### Sample collection

Mice were killed by decapitation, and the whole intestine was excised with a sterile scalpel under aseptic conditions, and the required intestinal segments were cut out, and the intestinal contents were scooped out and immediately placed on ice for division and labelling, and then loaded into sterilised centrifuge tubes according to the concentration of 0.2-0.5 g/tube and stored at 4℃, and DNA was extracted immediately on the same day.

### 16s rRNA sequencing

Fresh samples were immediately extracted for bacterial genomic DNA using a faecal DNA extraction kit (MP Biomedicals, USA) and stored at -80 °C according to the manufacturer’s protocol. The V3-V4 region of the bacterial 16 S rRNA gene was then amplified using the forward primer 338 F (5’-ACTCCTACGGGAGGCAGCA-3’) and the reverse primer 806R (5’-GGACTACHVGGTWTCTAAT-3’). PCR amplicons were purified using Agencourt AMPure beads (Beckman Coulter, Indianapolis, IN) and quantified using the PicoGreen dsDNA detection kit (Invitrogen). PCR products were then subjected to high-throughput pyrosequencing on an Illumina MiSeq platform at Shanghai Personal Biotechnology Co.

### Bioinformatic analysis

#### Analysis of microbial composition

Raw sequencing data were quality controlled, denoised, merged and chimeras removed using the QIIME2 software package. Amplicon sequence variation (ASV) signature sequences and ASV tables were then merged. The merged ASV table was diluted to calculate the specific microbial community composition at different taxonomic levels for each sample.

#### Analysis of microbial diversity

Diversity analyses were performed for H4 (4 h burn group), H6 (6 h burn group) and C (control group) using the QIIME2 and R software packages. For alpha diversity analysis, species richness was characterised using Chao-1 and observed species indices, and species diversity was characterised using Shannon and Simpson indices. For beta diversity analysis, Bray-Curtis distance was used to calculate the distance matrix, principal concordance analysis (PCoA) and non-metric multidimensional scaling analysis (NMDS) were used to assess differences between groups, Anosim was used to test the results, and the UPGMA algorithm was used for clustering.

#### Analysis of microbial variance

PCA analysis, Linear discriminant analysis Effect Size (LEFSe) analysis and Random Forest analysis were used to identify species that were significantly different between groups and to identify species that were discriminant. PCA was used to reduce the dimensionality of the data characteristics. In the LEfSe analysis, the Kruskal-Wallis rank sum test was used to determine the significance of differences between groups, followed by the Wilcoxon rank sum test to assess the differences of different features between subgroups, and finally linear discriminant analysis (LDA) was calculated. The Random Forest algorithm was used to build classifiers to determine the indicators and their significance in three data sets.

#### Microbial functional analysis

Phylogenetic Investigation of Communities by Reconstruction of Unobserved States (PICRUSt2) was used to predict the gene functions of significantly different species between groups. MetagenomeSeq was used to calculate significantly different pathways. STAMP (version 2.1.3) was used to identify differentially enriched KO modules between groups.

### Machine learning

Machine learning approaches were used to identify microbial species capable of differentiating acute burn phenotypes. Firstly, a random forest algorithm was used to calculate the contribution of microbial abundance to grouping and subfeatures were selected using a forward strategy. Secondly, four different machine learning algorithms including Random Forest, XGBoost, Naive Bayes and Support Vector Machine (SVM) were built for prediction. The performance was examined using leave-one-out (LOO) tests, and area under the curve (AUC) and confusion matrix were used to determine the best model.

### Statistical analysis

All differences between the means of the 3 groups were calculated using R software (version 4.2.2). Differences were considered statistically significant when *p* < 0.05.

## Results

### Successful establishment of deep burn models

Deep burns result in damage to the entire epidermal and dermal skin structure, which is highly susceptible to infection and can lead to shock and immune system problems. Mice are in the acute phase on day 1 post-burn and are physiologically hypermetabolised, so we performed rapid rewarming to prevent death of the mice. We observed the burned area compared to normal skin under the microscope (Olympus bx51T) by HE staining and found full thickness necrosis, collagen fibre disorganisation and vacuoles in the epidermis and dermis of the burned area. We also observed vasodilation and cognitive behavioural deficits in the burned group. These phenomena indicate that we have successfully established a mouse model of deep dermal burn and that sampling at 4 and 6 h ensured that samples were obtained in the acute phase.

### Statistics of microbiome sequencing results

Intestinal content samples were collected from 16 burned mice (H4 and H6) and 8 normal mice, and DNA was extracted for library construction, which was successful except for one sample in the H4 group. High-throughput 16s rRNA pyrosequencing was then performed on the 23 libraries, yielding a total of 30,281 ASV signature sequences. In terms of family, genus and species, an average of 1,654, 577 and 112 sequences were identified in the H4 group, an average of 1,991, 528 and 97 sequences were identified in the H6 group and an average of 1,088, 264 and 63 sequences were identified in the control group (Table S1 and Figure S1 A and B). These results were used for further analyses. The 23 microbiome datasets were counted at the phylum level, and it was found that the microbial composition of the first sample in the H4 group was significantly different from the other samples, and the number of ASVs was significantly lower than that of the other samples (Figure S1 C and D), which may be caused by insufficient DNA extraction. Therefore, we excluded data from this sample from the control analysis, but included it in the functional analysis and machine learning classification study.

### Deep burns lead to dysbiosis of the microbial community in the acute phase

**Fig. 1:**
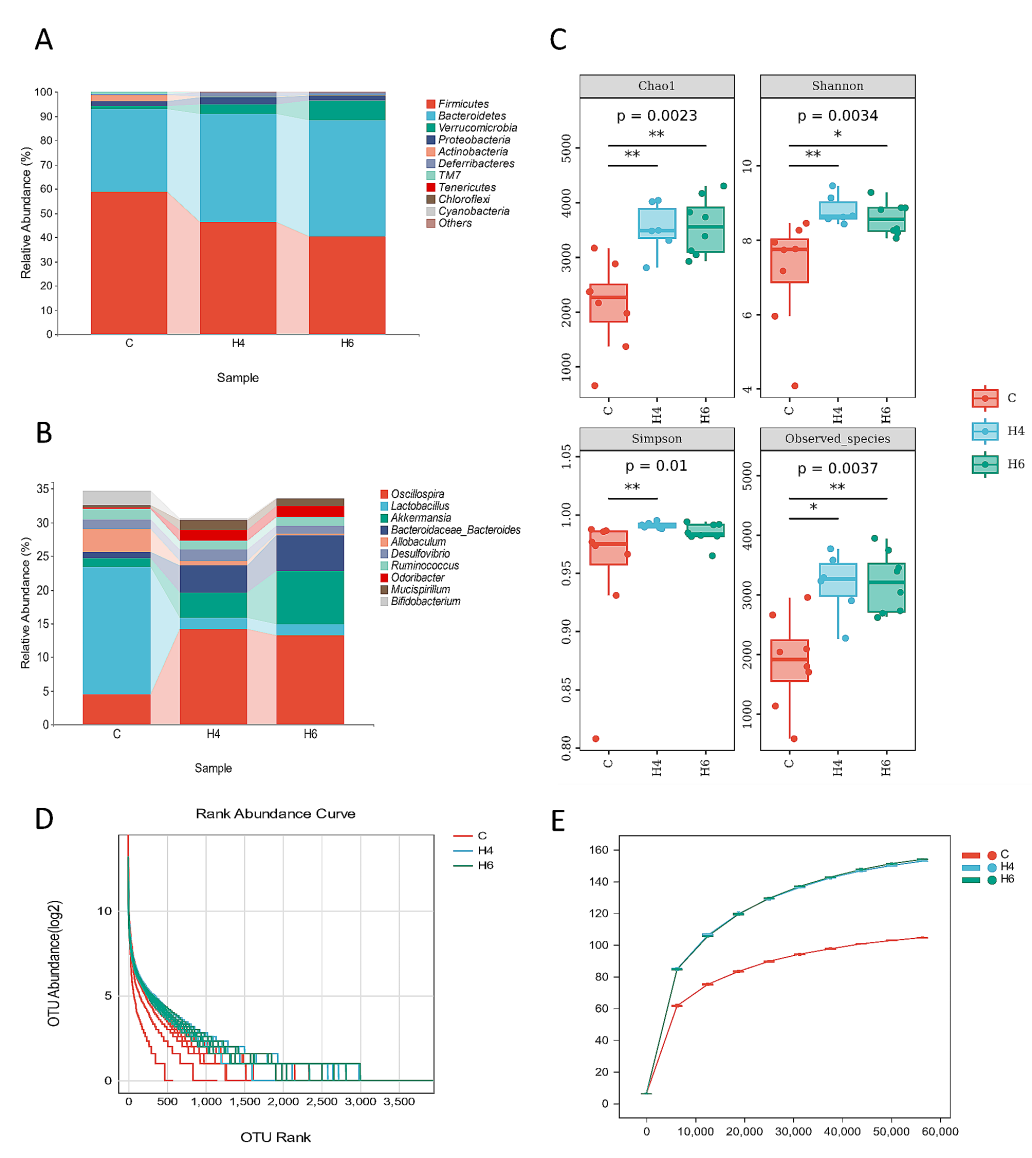
Analysis of the composition and alpha diversity of the intestinal flora of mice in the acute phase of severe burns. (**A**) Comparison of microbial composition at the phylum level (C: control, H4: 4-hours post-burn; H6:6 h post-burns); (**B**) Comparison of composition at the genus level; (**C**) Comparison of α diversity indices between groups ; (**D**) microbial abundance curves ;E) microbial rarefaction curve

At the phylum level, the major bacterial phyla showed clear trends at 4 and 6 h post-burn. The abundance of the phylum Firmicutes and Actinobacteria declined rapidly at 4 h post-burn, and then further at 6 h (*p* < 0.05). The opposite trend was observed for Bacteroidetes, Verrucomicrobia and TM7, whose relative abundance continued to increase at both 4 and 6 h (*p* < 0.05, Fig. 1A). The Firmicutes/Bacteroidetes (F/B) ratio was 1.71 in the control group, while it decreased to 1.05 and 0.95 in the H4 and H6 groups, respectively (Figure S1B). The abundance of another major bacterial phylum, Proteobacteria, showed little fluctuation. At the genus level, the H4 and H6 groups had similar compositions, with no significant differences observed except for Akkermansia (*p* < 0.05). Compared with the control group, the relative abundances of Oscillospira, Bacteroidaceae_Bacteroides, Akkermansia, Odoribacter, and Mucispirillum were significantly up-regulated after the burn injury, while the abundances of Lactobacillus, Allobaculum and Bifidobacterium were significantly down-regulated (*p* < 0.05). The abundance of Akkermansia continued to increase after the burn injury, with the H6 group showing a significant increase compared to both the H4 group and the control group; while the Desulfovibrio remained essentially unchanged across the three groups (Fig. 1B). The ASV/OTU Venn diagram clearly illustrates the different numbers of ASVs/OTUs between the control group, the 4 h post-burn group, and the 6 h post-burn group.

We compared the α-diversity indices of the gut microbiota among the 23 groups. As shown in Fig. 1C, significant differences (*p* < 0.05) were observed in chao1, Simpson, observed-species, and Shannon indices between the pre-burn and post-burn samples. However, the differences in these indices between the 4-hour and 6-hour post-burn samples were relatively small. The microbial abundance curves suggested that higher number of ASVs were detected in the burn group than in the control group(Fig. 1D). In the rarefaction curve, we observed a significant increase in the diversity of the gut microbiota after the burn injury, with a slight increase in diversity as the acute burn phase progressed. The results reached a stable state when the sequencing depth reached around 30,000 (Fig. 1E).

**Fig. 2:**
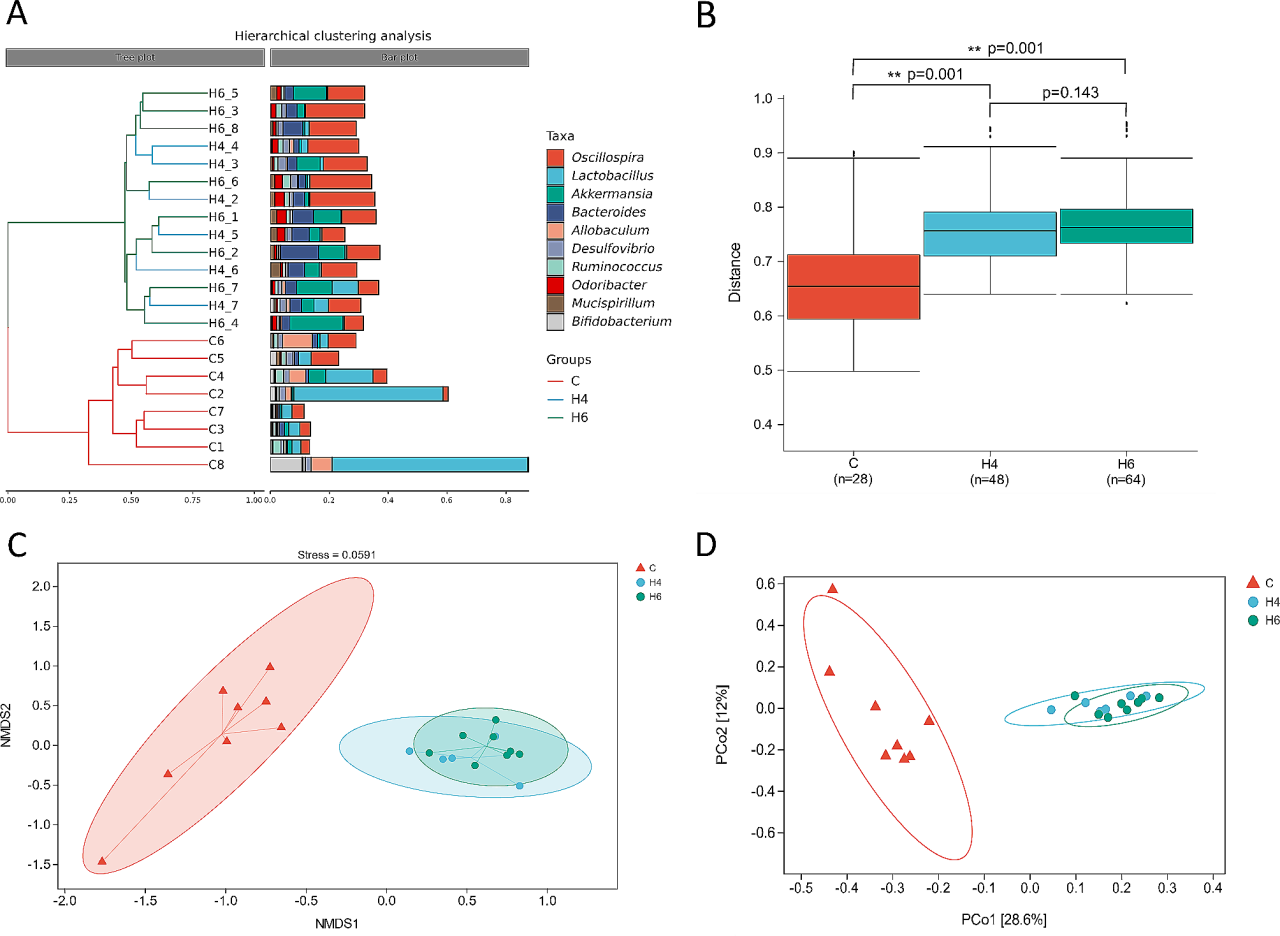
Analysis of differences in the gut microbiome between the burn and control groups. (**A**) Heuristic clustering of 22 mice gut microbiome samples (group C: C1-C8, group H4: H4_2-H4_7; group H6: H6_1-H6_8); (**B**) Boxplots of microbial abundance in burn and control groups; (**C**)NMDS analysis of the gut microbiome between the burn and control groups; (**D**) PCoA analysis of the gut microbiome between the burn and control groups

For β diversity, we performed a PCOA analysis, which revealed a clustering tendency between samples in the distance matrix (Fig. 2A and D). Hierarchical cluster analysis showed clear separability between the control and burn groups (Fig. 2A), indicating significant changes in gut microbiota species richness before and after burn injury. The largest difference was observed between the 6 h post-burn group and the control group, indicating an acute response of the mice to the burn injury. ANOWA analysis showed no significant difference between H4 and H6 (*p* = 0.143) (Fig. 2B), but both were significantly different from the control group (*p* < 0.05). We also performed NMDS analysis (Fig. 2C) and found that the differences in microbial community clustering between pre- and post-burn samples were more significant than those observed in PCoA, with a stress value much lower than 0.2, indicating the reliability of the analysis results. These results indicate an increase in species richness and diversity of gut microorganisms during the acute phase of burns, in contrast to other reports that indicate a decrease in diversity.

### Characterisation of microbial changes in the acute phase after burns

**Fig. 3:**
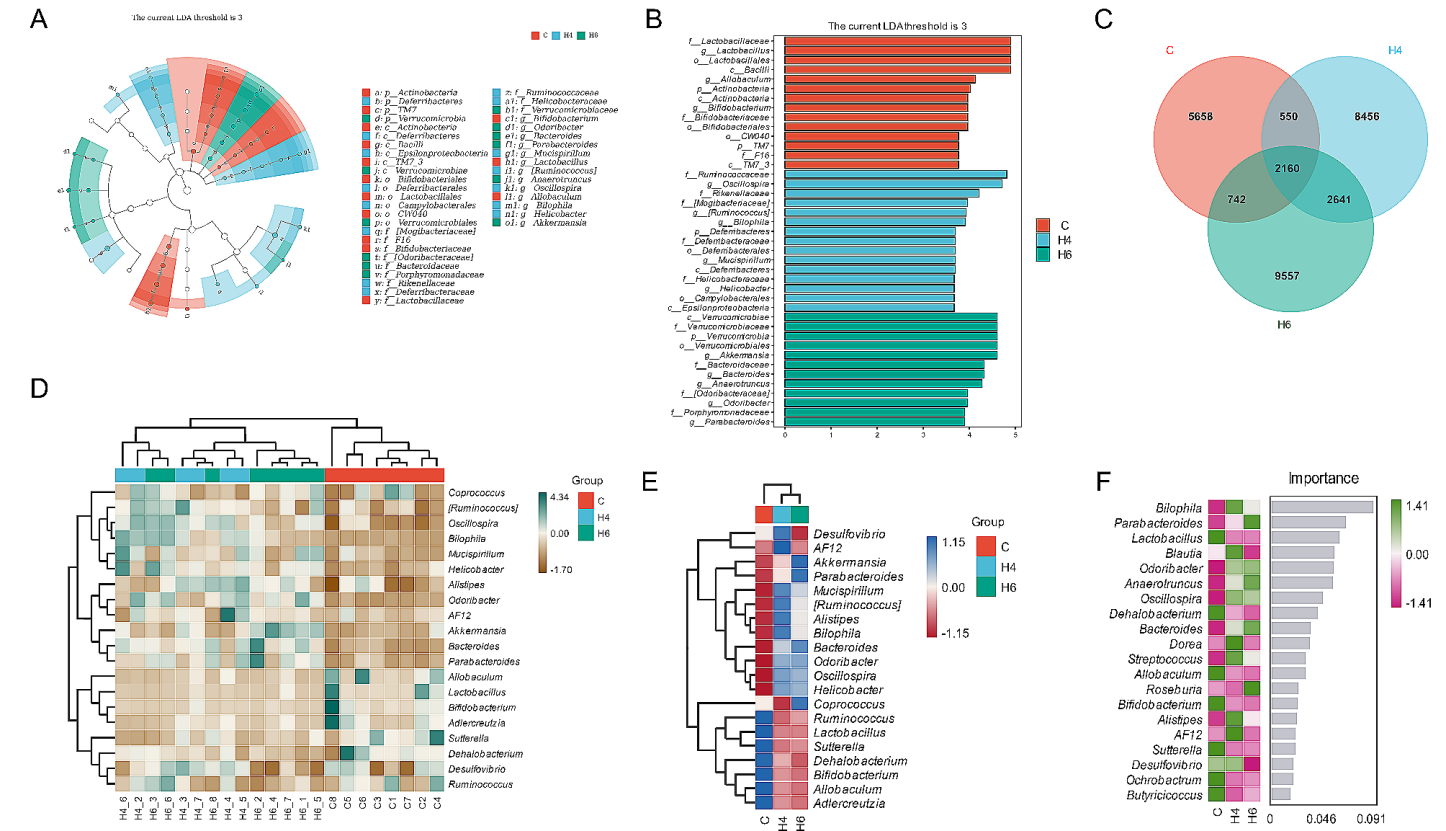
Analysis and screening of differential microorganisms between burn and control groups. (**A**) Phylogenetic trees of significantly different species obtained by LEfSe(LDA Effect Size) analysis (LDA ≥ 3.0); (**B**) Histogram of significantly different species obtained from LEfSe analysis; (**C**) Venn diagram of average microbial population obtained by sequencing of three groups; (**D**) Heatmap of the intestinal flora of 22 samples at the genus level (wards.D2 algorithm was used for both species clustering and sample clustering); (**E**) Heatmap of the intestinal flora of the burn and control groups at the genus level (wards.D2 algorithm was used for both species clustering and sample clustering); (**F**) Ranking of importance of bacterial genus for classifying three groups (Random Forest algorithm)

The LEfSe analyses (Fig. 3A and B) revealed several significantly different bacteria between the burn and control groups. Taxonomically, bacteria from several genera of the phylum Campylobacterota, the phylum Deferribacterales, the phylum Verrucomicrobiota, the order Bacteroidales, and the order Eubacteriales were significantly increased after burn injury, including four genera from group H4: Oscillospira Ruminococcus, Bilophila, Mucispirillum and Helicobacter, and five genera from group H6: Akkermansia, Bacteroides, Anaerotruncus, Odoribacter and Parabacteroides. Multiple groups of bacteria from phylum Actinobacteria, phylum TM7, and class Bacilli are more abundant in normal mice, particularly at the genus level including Lactobacillus, Allobaculum, and Bifidobacterium. Species crossover Venn diagrams also show differences in the sequences detected on the three sets of samples, with the sequences shared between the two burn groups far exceeding those shared between them and the control group (Fig. 3C).

The samples and groups were clustered using the 20 most distinct bacteria at the genus level, and the resulting heat maps are shown in Fig. 3D and E. They clearly distinguish burned mice from normal mice and define the three groups. These results are consistent with the results of the LEfSe analysis and can be divided into three cases. The first are those with a significant decrease in abundance after burns, such as Ruminococcus, Lactobacillus, Sutterella, Dehalobacterium, Bifidobacterium, Allobaculum, Adlercreutzia; the second are those with a significant increase in abundance after burns, such as Akkermansia, Parabacteroides, Mucispirillum, Ruminococcus, Alistipes, Bilophila, Bacteroides, Odoribacter, Oscillospira, Helicobacter; and the third are those with a sharp increase at 4 h after burns followed by a recovery starting from 6 h onwards, such as Desulfovibrio and AF12. We used a random forest model to determine the importance of different bacteria in distinguishing between the three groups of samples, and the top bacteria included Bilophila, Parabacteroides, Lactobacillus, Blautia, Odoribacter, Anaerotruncus, and others (Fig. 3F).

### Response of microbial functions after burn injury

**Fig. 4:**
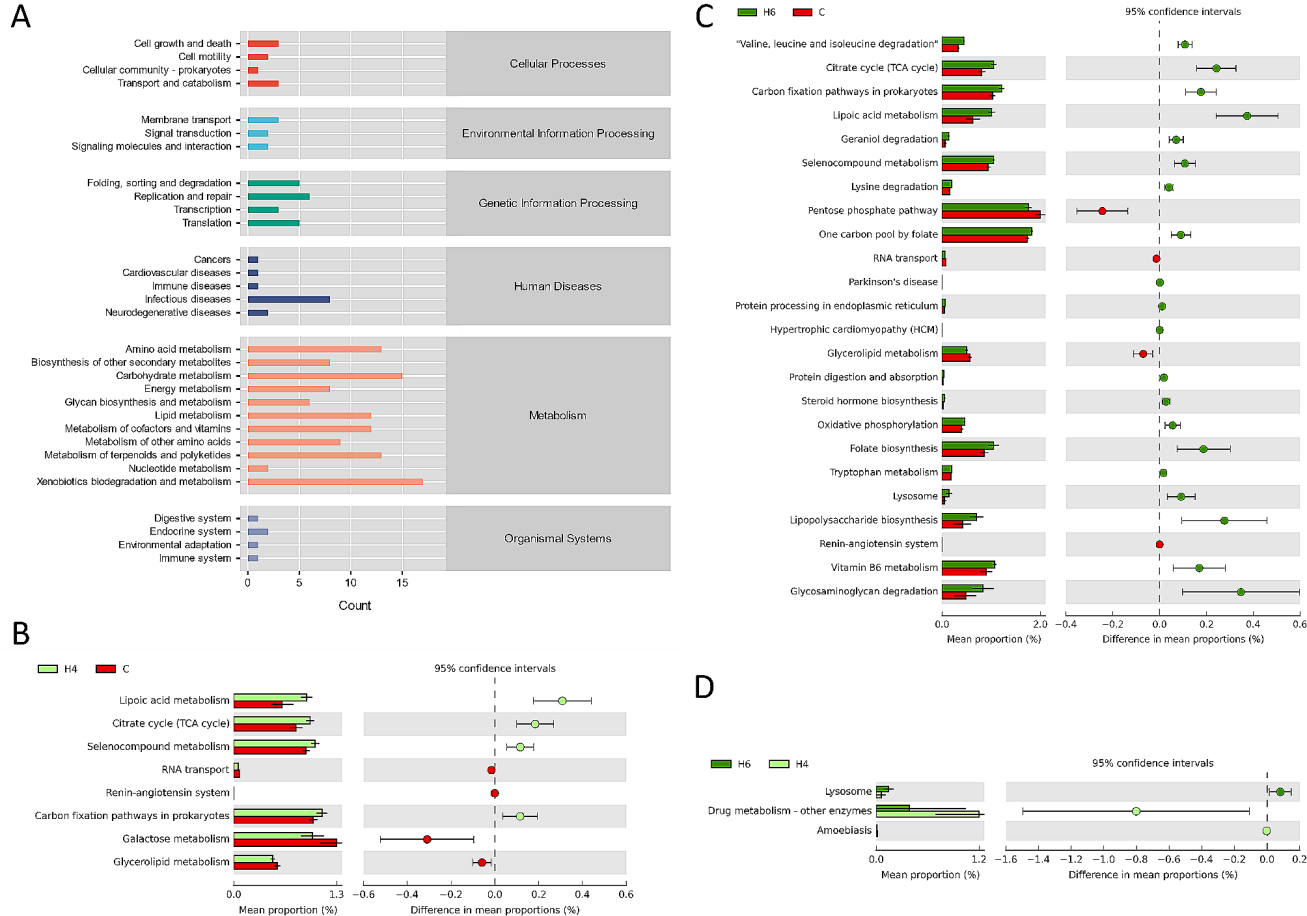
Function and pathway analysis of differential bacteria in burn and control groups. (**A**) Metabolic pathways of differential bacterial enrichment between three groups (by number of species); (**B**) Significantly different metabolic pathways and 95% confidence intervals between the 4-hour post-burn group and the control group; (**C**) Significantly different metabolic pathways and 95% confidence intervals between the 6-hour post-burn group and the control group; (**D**) Significantly different metabolic pathways and 95% confidence intervals between the 4-hour post-burn group and the 6-hour post-burn group

We extrapolated the functions of differential bacteria to determine which functional adjustments were primarily involved in the changes in gut microbiology after burn injury. We found that the bacteria that changed after burn injury were mainly enriched in several classes of metabolic processes and also involved in functions such as repair, transcription, cell growth and death; in terms of diseases, they were mainly involved in infection, immunity and neurological damage (Fig. 4A). Specifically, lipoic acid metabolism, citrate cycle, seleno-compound metabolism and carbon fixation pathway were enhanced, whereas galactose metabolism, glycerolipid metabolism were weakened in the acute period after burns (Fig. 4B and C). In the 6-hour burn group, amino acid degradation, folate biosynthesis, lipopolysaccharide biosynthesis, vitamin B6 metabolism, and glycosaminoglycan degradation were also enhanced, and the pentose phosphate pathway was weakened (Fig. 4C). In contrast, only lysosome and drug metabolism differed between the H4 and H6 groups (Fig. 4D).

### Machine learning models accurately discriminated burn phenotypes

**Fig. 5:**
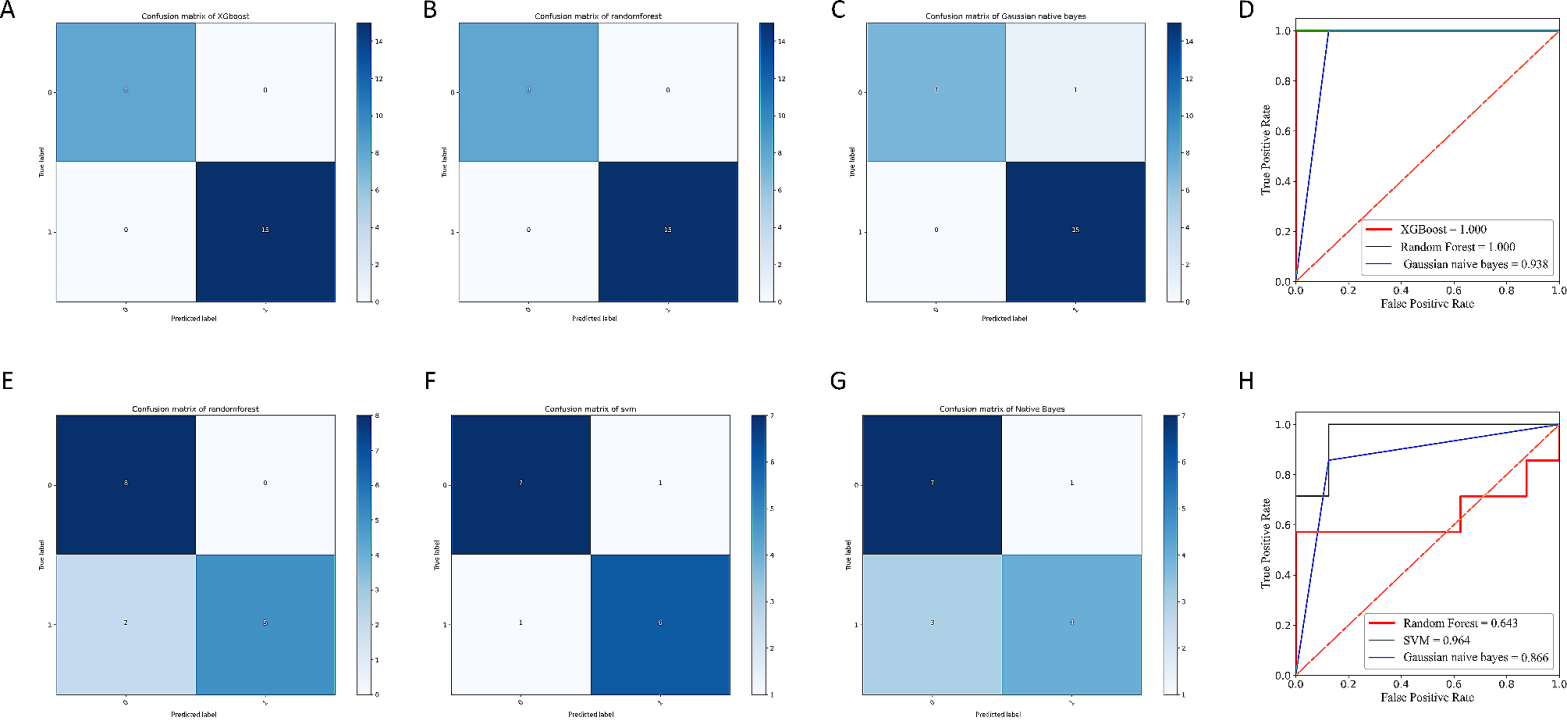
Results of machine learning classification of three groups of samples. (**A**) Confusion matrix for the classification of burn and control groups using the XGBoost algorithm; (**B**) Confusion matrix for the classification of burn and control groups using the Randomforest algorithm; (**C**) Confusion matrix for the classification of burn and control groups using the Gaussian Naive Bayes algorithm; (**D**) ROC curves for classification of burn and control groups; (**E**) Confusion matrix for the classification of 4-hour post-burn group and the 6-hour post-burn group using the Randomforest algorithm; (**F**) Confusion matrix for the classification of 4-hour post-burn group and the 6-hour post-burn group using the SVM algorithm; (**G**) Confusion matrix for the classification of 4-hour post-burn group and the 6-hour post-burn group using the NaiveBayes algorithm; (**H**) ROC curves for classification of two burn groups

In the model building process, the top twenty biomarkers were first obtained, and then classification was performed. Both XGBoost and Random Forest achieved 100% accuracy in classifying the control and burn groups (Fig. 5A-D). SVM achieved 86.7% accuracy in classifying the 4 and 6 H groups (Fig. 5E-H). These results demonstrate that it is possible to predict the symptoms and severity of burn injury in mice by using specific microbial abundances as feature values. The slightly lower prediction accuracy for Burn 4 H and Burn 6 H is due to the fact that both time points are in the acute phase of burn when changes in the microbial community are minimal. However, these trends can still be observed.

## Discussion

Severe burns are horrific acute traumatic injuries that can lead to serious complications such as sepsis, multi-organ failure and high mortality rates worldwide. The gut flora plays an important role in this pathogenic process. Dysbiosis of the gut flora after burns leads to impaired nutritional and metabolic functions of the microorganisms. In addition, bacteria are transferred to the bloodstream and other organs of the body, which is associated with many of the serious complications. However, there is currently a lack of understanding of the changing characteristics of gut microbes in the early stages of severe burns and potential markers. Therefore, we undertook this study to establish mouse models of deep burn injury, to investigate how the gut flora of mice is altered during the acute phase of the first day of burn injury, and to try to uncover markers that are closely associated with burn phenotypes.

Several published studies have shown a significant reduction in the diversity of the gut flora and a decrease in the number of species after severe burns. However, our study contradicts this conclusion. Both Venn diagrams, sparse curves, and various alpha diversity indices strongly suggest that the diversity of gut microorganisms is increased and significant within 24 h of severe burns, while the major bacterial species are relatively similar. The results of Caldwell et al. are consistent with our conclusions [7]. The results of the beta diversity analyses further demonstrated that the gut microbial community of mice was significantly altered after burn injury. Heuristic cluster analysis, PcoA analysis and NMDS analysis all clearly separated the burned group samples from the unburned group samples, with the NMSD stress value reaching a very low 0.0591. ANOVA analysis based on Bray-Curtis distance further revealed that the difference in flora between the burned and control groups was highly significant, but not significant between the two groups of burned samples.

Similar to previous reports [7, 15], we observed an overgrowth with normally low- abundant gram-negative bacteria of the Proteobacteria, Deferribacteres, Verrucomicrobia and Bacteroidetes phyla, compared to the normal predominance of gram-positive Firmicutes. In conclusion, the available findings suggest that this trend occurs within the first day after the burn injury and is the main contributor to the increase in the diversity index. Over time, the diversity will decrease and then return to normal. We have seen the growth of the genera Oscilibacter, Escherichia, Mucispirillum, Bacteroides, and Akkermansia, which comprise a wide range of opportunistic pathogens and are important causes of nosocomial bloodstream infections. For example, Mucispirillum may migrate from the mucus layer to the intestinal luminal compartment as a result of post-burn intestinal permeability, completing the critical transition that triggers colitis [16]. We also noted a decrease in potentially protective bacteria. Most butyrate-producing bacteria belong to the phylum Firmicutes and were significantly lower in burned mice. In particular, at the genus level, bacteria involved in digestion and metabolism in vivo, such as Lactobacillus, Allobaculum, and Bifidobacterium, were reduced.

The phylum Firmicutes and Bacteroidetes are the two most dominant groups of bacteria in the intestinal microbiome. It is widely accepted that the Firmicutes/Bacteroidetes (F/B) ratio has an important influence on the maintenance of normal intestinal homeostasis [17]. An increased or decreased F/B ratio is considered dysbiosis, with the former commonly observed in obesity, and the latter in inflammatory bowel disease (IBD) [18]. We observed a sustained decrease in F/B at 4 and 6 h post-burn (Table S2 and Figure S1B), suggesting that the burn may have initiated an inflammatory response that progressively intensified.

In addition to elucidating the bacteria that differed between groups at the phylum and genus level, we also examined differences at the species level to further define the functions of these bacteria (Figure S2 A and B). The results of the principal component analysis also supported the previous conclusion of a significant difference between the burn and control groups, further demonstrating the validity of the groupings (Figure S2 C). Bacteria of the genus Lactobacillus are a class of probiotics in the gut flora that promote digestion, inhibit inflammatory mediators, and enhance immunity. At least four species of Lactobacillus showed a significant decrease in abundance at 4 and 6 h post-burn, further confirming that both digestive and anti-inflammatory functions are compromised after burn injury. Additionally, a number of well known probiotics such as Selenomonas_lacticifex, Bifidobacterium, Prevotella_copri, Clostridium_celatum, Butyricicoccus_pullicaecorum, Faecalibacterium_prausnitzii, etc. were found to be reduced in the burn group. These bacteria are important participants in the digestion of sugars, proteins, and plant fibres, or are essential for maintaining the integrity of the intestinal mucosa to prevent invasion by pathogens, or have immune-boosting and antioxidant effects.

Bacteria with increased abundance in the burn group played a more complex role. There was a significant increase in the relative abundance of Ruminococcus_gnavus and Bacteroides_acidifaciens, which have been found to be associated with reduced immunity and tumour growth [19]. Some opportunistic pathogenic or rare bacteria such as Mucispirillum_schaedleri, Alistipes_massiliensis, Streptococcus_alactolyticus, Desulfovibrio_alaskensis, Staphylococcus_sciuri, and Clostridium_methylpentosum, which are consistently increased after burns, are responsible for driving the development of IBD [20–22]. The increase in Bacteroides_uniformis, Staphylococcus_sciuri, and Ruminococcus_flavefaciens is thought to potentially lead to psychosis, depression, and cognitive decline, suggesting that burns may perhaps be detrimental to cognitive abilities [23, 24]. We also observed a gradual increase in the levels of Akkermansia_muciniphila and Parabacteroides_distasonis from group H4 to H6, which have been reported to be associated with intestinal metabolism, controlling metabolic levels to prevent the onset of obesity and may also contribute to gastrointestinal disorders [25, 26].

Metabolic pathway analysis has shown that after burn injury in mice, there is a significant decrease in lactose metabolism, leading to lactose metabolism disorders. This can lead to the accumulation of lactose in the body, potentially causing lactosemia, which can lead to liver and brain damage, resulting in neural impairment and cognitive deficits. There is also an increase in the TCA (tricarboxylic acid) cycle and sulphur amino acid metabolism, while glycerol metabolism decreases. This indicates an increased energy requirement and accelerated metabolism leading to a state of hypothermia. The increased levels of redox carriers and enhanced seleno-compound metabolism result in a greater ability to regulate inflammation. In addition, the presence of post-burn infection promotes microbial growth and enhances carbon fixation by anaerobic organisms, leading to reduced glycerol metabolism. Comparing the 6-hour (6 H) group to the 4-hour (4 H) group, there is a further decrease in the ability of the mice to metabolise glucose-6-phosphate, indicating a more profound effect on TCA metabolism. There is also a reduction in 5-phospho-ribose, which can affect RNA. The synthesis of folate is increased, which is linked to the regulation of neuronal cell development. Lipopolysaccharide(LPS) synthesis increases, indicating the activation of the body’s immune system to control inflammation. The main difference between the 4 and 6 H groups is an increase in lysosome content and a decrease in enzymes, which can lead to microbial growth and disruption of intestinal homeostasis. In conclusion, changes in the microbiota following burns affect lactose and glucosaminoglycan metabolism, and in combination with associated changes in harmful bacteria, this is likely to indirectly affect brain cognition.

The machine learning results showed that the screened characteristic bacteria are potential biomarkers for distinguishing early severe burns. XGBoost and Random Forest are worthwhile algorithms when using these bacteria to predict phenotypes. Specifically, the increased abundances of Bilophila, Parabacteroides, Odoribacter, Oscillospira, Anaerotruncus, Bacteroides may indicate a rapid progression during the acute inflammatory phase of burns. On the other hand, the abundances of probiotics such as Lactobacillus, Dehalobacterium, Bifidobacterium, Allobaculum, Sutterella, Ochrobactrum, and Butyricicoccus continues to decline after burns and are potential biomarkers of impaired metabolic capacity. Besides, to infer the time periods in early severe burns, the abundance of Desulfovibrio, Clostridium, Campylobacter could be considered as predictors.

Limitations of this study include a limited sample size and a limited number of selected time points, so the microbial changes revealed in the serve burn injuries may not fully capture the comprehensive features. Besides, the machine learning results can only be informative and cannot demonstrate the generalisability of the model to large datasets. A further limitation is the lack of studies on burn outcome parameters.

## Conclusions

Our study revealed trends in the gut microbiota during the acute phase of severe burns and showed that the main alterations in gut flora during this phase are characterised by a decrease in probiotics and an increase in bacteria associated with inflammation and cognitive impairment. Additionally, the development of a machine learning model to differentiate burn status using signature microbes provides a new perspective on understanding dysbiosis in the gut microbiota associated with burns. This offers a potential avenue for adjunctive detection and diagnosis of burn severity.

### Electronic supplementary material

Below is the link to the electronic supplementary material.

Supplementary Material 1

## Data Availability

Raw data from all 23 mice with gut microbiome sequencing are freely available in the NCBI SRA database under BioProject PRJNA1023023, with sample accession numbers SRR26249944 ~ SRR26249966.

## Abbreviations

PTSD: post-traumatic stress disorder
TCA: cycle tricarboxylic acid cycle
XGboost: eXtreme Gradient Boosting
ASV: amplicon sequence variation
OTU: operational axonomic unit
PCoA: principal concordance analysis
PCA: principal component analysis
LDA: linear discriminant analysis
NMDS: non-metric multidimensional scaling analysis
UPGMA: unweighted pair-group method with arithmetic means
LEfSe: linear discriminant analysis Effect Size
PICRUSt: Phylogenetic Investigation of Communities by Reconstruction of Unobserved States
SVM: support vector machine
LOO: leave-one-out
AUC: area under the curve
