# Supplementary material for: In silico analysis of intestinal microbial instability and symptomatic markers in mice during the acute phase of severe burns
Source: BMC Microbiol. 2024 Apr 15;24:124. doi: 10.1186/s12866-024-03266-9 (PMC11017597; doi:10.1186/s12866-024-03266-9)
Supplement: Supplementary file 1 — Supplementary Material 1 [file 12866_2024_3266_MOESM1_ESM.pdf]

Supplementary Materials

Table S1 Species count of 23 sequenced samples

ID	domain	phylum	class	order	family	genus	species	unclassified
C1	18	0	1	559	1236	208	64	0
C2	23	2	5	222	595	251	54	0
C3	33	1	4	504	2082	257	67	0
C4	37	4	1	349	1034	305	61	0
C5	12	1	3	441	876	290	69	0
C6	16	2	2	480	1130	340	60	0
C7	21	0	2	728	1568	274	73	0
C8	24	0	1	137	179	185	58	0
H4_1	29	11	111	299	531	625	209	0
H4_2	58	1	0	989	1837	773	106	0
H4_3	48	2	0	423	1806	534	92	0
H4_4	22	2	0	399	1290	471	69	0
H4_5	32	0	1	423	2281	468	94	0
H4_6	47	0	0	785	1733	577	106	0
H4_7	36	4	5	744	2101	588	110	0
H6_1	25	0	3	379	1729	468	84	0
H6_2	20	2	2	482	1556	477	88	0
H6_3	29	0	0	773	1467	658	102	0
H6_4	38	0	2	617	2212	419	87	0
H6_5	34	0	1	570	1608	425	100	0
H6_6	51	1	2	650	2182	749	100	0
H6_7	54	6	3	525	2746	488	128	0
H6_8	27	0	3	366	2427	541	86	0

Table S2 Abundance statistics for the top 10 bacterial phylum of 23 sequenced samples

ID	H4_1	H4_2	H4_3	H4_4	H4_5	H4_6	H4_7	C1	C2	C3	C4	C5	C6	C7	C8	H6_1	H6_2	H6_3	H6_4	H6_5	H6_6	H6_7	H6_8
Firmicutes	0.05652894	0.63141293	0.40752456	0.49743872	0.25552019	0.45821238	0.52448308	0.63417248	0.66154493	0.32670365	0.44204354	0.70765658	0.52645586	0.60254132	0.80783239	0.27985625	0.3707695	0.568407	0.33779791	0.4311265	0.54244516	0.36231379	0.34139309
Bacteroidetes	0.11910845	0.31204765	0.45877671	0.45090288	0.65181143	0.42334526	0.37727575	0.32653338	0.26748292	0.6317689	0.4411319	0.22409941	0.4266203	0.37121616	0.01447493	0.58716175	0.51020318	0.36602208	0.46050053	0.4157796	0.39303574	0.48287926	0.61477496
Proteobacteria	0.4843765	0.02275347	0.03941576	0.03172951	0.02698196	0.02939809	0.02694606	0.01444538	0.02891224	0.01500347	0.03573848	0.02693611	0.02159149	0.00857473	0.02294207	0.01351678	0.01343762	0.03348179	0.0089339	0.01290927	0.0383341	0.02008897	0.02501691
Verrucomicrobia	0.00924689	0.01409082	0.08058074	0.00741486	0.03767706	0.05124216	0.04308281	0.01458102	0.00537047	0.01528715	0.0592808	0.00018828	0.00928936	0.00696049	0.00028107	0.09448333	0.09037314	0.02559007	0.18130557	0.11410645	0.00642737	0.12142032	0.00782886
Fusobacteria	0.28842803	0	0	0.00002339	0	0	0	0	0	0.00029944	0.00002464	0	0	0	0	0	0	0	0	0	0	0.00022163	0
Actinoba	0.0116	0.0006	0.0046	0.0049	0.0024085	0.0015	0.0162	0.0059	0.0213	0.0023	0.0151	0.0253	0.0074	0.0035	0.1255	0.0007	0.0007351	0.0007	0.0057	0.0019	0.0012	0.0044	0.0021

cteria	8422	4049	6459	8222		0759	6029	9517	9779	1671	5282	6714	3149	3948	5481	5093		5436	3815	1836	2718	0089	7468
Deferribacteres	0.00113314	0.01437904	0.00485409	0.00464306	0.02083439	0.03343972	0.00660912	0.00119361	0.00133473	0.0050747	0.00128121	0.01171081	0.00477021	0.00275458	0.00005856	0.02199158	0.01240848	0.00224856	0.00178962	0.02198183	0.01441939	0.00360937	0.00745836
TM7	0.00045967	0.00032025	0.00141395	0.00145022	0.00069055	0.00019246	0.00205377	0.00150557	0.01180242	0.00208031	0.00289505	0.00342663	0.00293745	0.00336177	0.02642026	0.00041569	0.00066159	0.00129111	0.00059654	0.0005481	0.00101243	0.0011398	0.00045105
Tenericutes	0.00963173	0.00144111	0.00071426	0.00052629	0.00168427	0.00004811	0.0003706	0.0001492	0.00070415	0.00018912	0.0003819	0.00006276	0.00022596	0.00028138	0.00103058	0.00033524	0.00011762	0.00060929	0.00065335	0.00027405	0.00029146	0.00083902	0
Cyanobacteria	0.00920413	0.00009607	0	0.00007017	0.00021895	0.00003208	0.00006177	0.00008138	0.00056752	0	0.00002464	0.00022593	0.00010043	0.00001481	0.0001054	0.00005364	0.0000294	0.00005803	0	0.00004327	0.00006136	0.00033244	0
Others	0.0101983	0.00281816	0.00205533	0.00081868	0.0021727	0.00258216	0.00285675	0.00134281	0.00088282	0.00127656	0.00204501	0.00032635	0.00057745	0.00075529	0.00129993	0.00143482	0.00126437	0.00153773	0.00268443	0.00131256	0.00274582	0.00275452	0.00090209

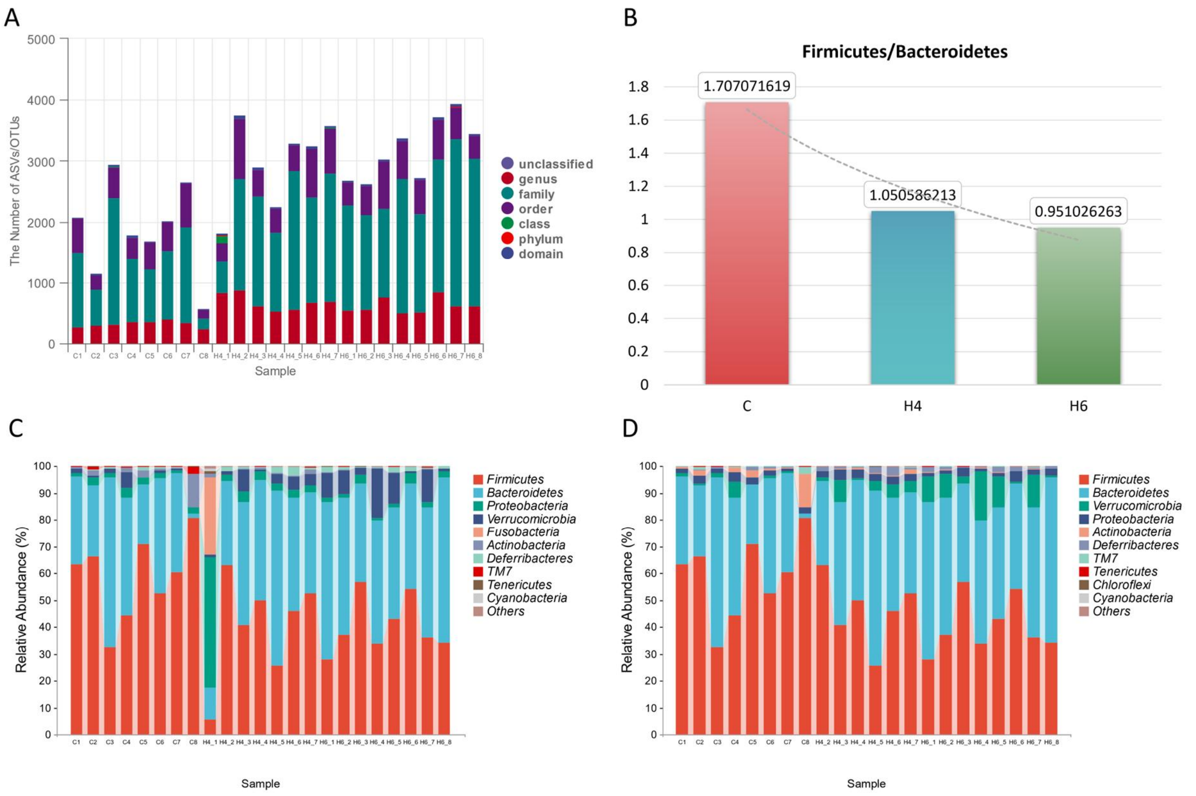

Figure S1 Distribution of species at the phylum level in 23 samples

A) Histogram of gut microbial distribution at different taxonomic levels in 23 samples; B) Firmicutes/Bacteroidetes ratios for the three sample groups; C) Histogram of microbial composition at the phylum level for the 23 sequenced samples; D) Histogram of microbial composition at the phylum level for the 22 sequenced samples (excluding H4_1)

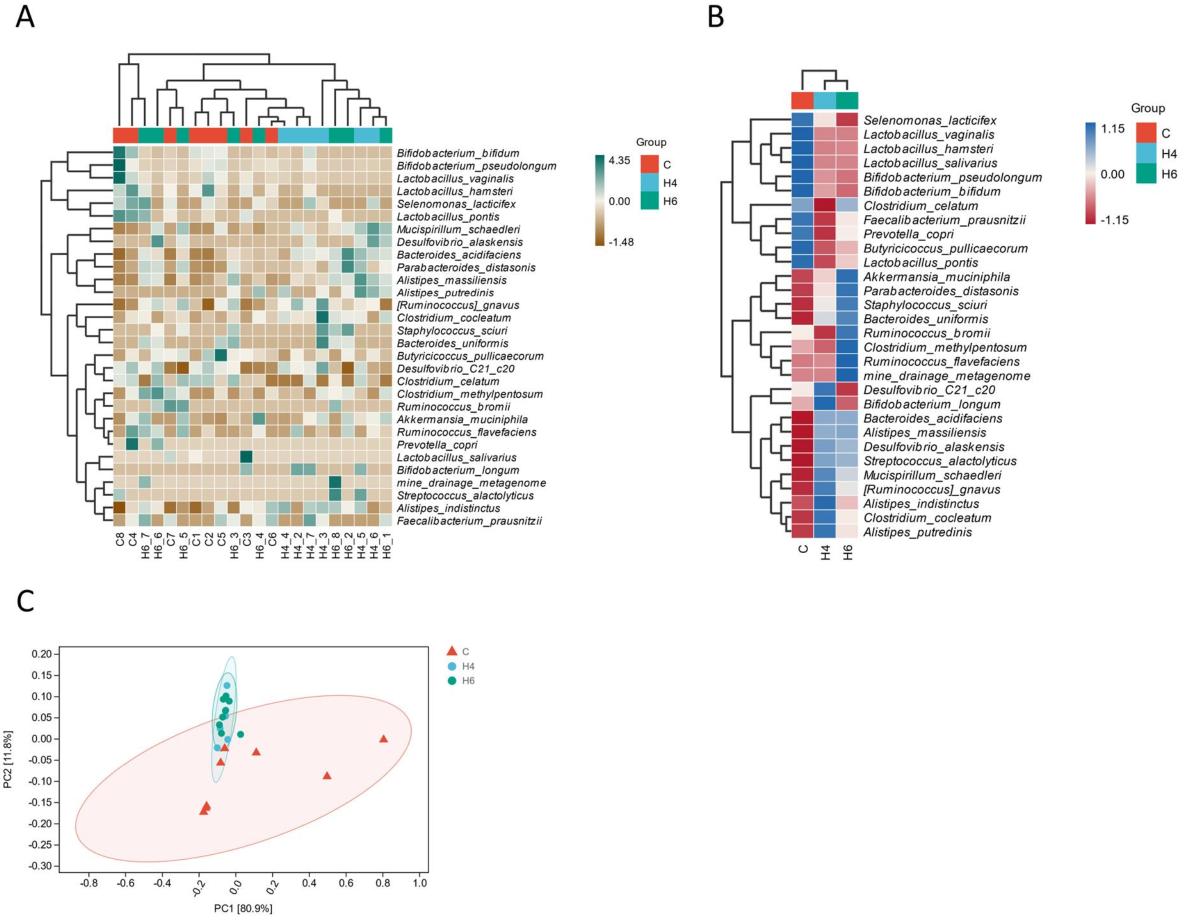

Figure S2 Heatmap of sample clustering at species level and OPLA-DA clustering results

A) Heatmap of the intestinal flora of 22 samples at the species level (wards.D2 algorithm was used for both species clustering and sample clustering); B) Heatmap of the intestinal flora of the burn and control groups at the species level (wards.D2 algorithm was used for both species clustering and sample clustering); C) OPLS-DA analysis of differences between the three groups
